# Assessment of metabolites in urine in post-kidney transplant patients: insights into allograft function and creatinine clearance

**DOI:** 10.1007/s11306-025-02246-y

**Published:** 2025-03-27

**Authors:** Eva Baranovicova, Matej Vnucak, Karol Granak, Patricia Kleinova, Erika Halasova, Ivana Dedinska

**Affiliations:** 1 Biomedical Centre BioMed, Jessenius Faculty of Medicine, Comenius University in Bratislava, Mala Hora 4, 036 01 Martin, Slovakia; 2 Transplant-Nephrology Department and 1st Internal Department, University Hospital Martin, Jessenius Faculty of Medicine, Comenius University in Bratislava, Mala Hora 4, Martin, Slovakia

**Keywords:** Kidney transplantation, Metabolomics, Urinary metabolites, Allograft function, Creatinine clearance, NMR spectroscopy, Renal function assessment

## Abstract

**Introduction:**

The suboptimal function of transplanted kidney can lead to imbalances in processes controlled by the kidneys, necessitating long-term monitoring of the graft’s function and viability. Given the kidneys’ high metabolic activity, a metabolomics approach is well-suited for tracking changes in post-transplant patients and holds significant potential for monitoring graft function.

**Objectives:**

Examination of the response of urinary creatinine levels to (i) serum creatinine levels and (ii) allograft function during periods of impaired kidney function in post-transplant patients.

**Methods:**

We analyzed morning and 24-h urine samples from 55 patients who underwent primary kidney transplantation and were uniformly treated with immunosuppressants, with an average follow-up of 50 months post-surgery. We assessed the relative levels of urinary metabolites detectable by NMR spectroscopy and investigated correlations between these metabolite levels and renal function.

**Results:**

We observed rather unexpected independence of urinary creatinine levels on levels of serum creatinine as well as on allograft function expressed by eGFR (estimated glomerular filtration rate). This observation allowed a very good agreement of outcomes from raw and creatinine-normalized data, consistent for both morning urine samples and 24-h urine collections. The urinary levels of citrate and acetone were detected to be sensitive to allograft function, and the urinary levels of metabolites in combination showed promising prediction for kidney function, on the level of p-value: for 24 h pooled urine: 4.6 × 10^−12^ and morning urine: 5.36 × 10^−9^. We discussed the data also in the light of metabolic changes in blood plasma.

**Conclusion:**

We support the opinion of critical assessment of renal creatinine clearance when judging the filtration function of the allograft. As the next, urinary metabolomics can serve as an easily available supplement to prediction for allograft function in patients after kidney transplantation.

**Supplementary Information:**

The online version contains supplementary material available at 10.1007/s11306-025-02246-y.

## Introduction

Kidneys are the second most metabolically active organs in the body. When renal function is impaired, it disrupts several critical processes, including acid–base balance, electrolyte and fluid regulation, hematopoiesis, waste excretion, and the exchange of nitrogenous metabolites between organs (Dejong et al., 1993; 1996; van de Poll et al., 2004). Moreover, a poorly functioning kidney creates metabolic challenges for the body, which can result in compensatory effects as well as secondary damage (Slee, 2012). For patients with final-stage kidney disease, to restore impaired functions and improve both survival and long-term outcomes, kidney transplantation is the preferred treatment. Although kidneys from living donors typically start functioning immediately, their performance can be suboptimal and requires careful monitoring throughout the post-transplant period. As a result, transplant specialists focus on identifying new noninvasive indicators that can predict or correlate with graft function to enhance patient care.

Metabolomic research in renal transplantation has yielded valuable insights. Effective metabolic recovery is a key determinant of post-transplant outcomes (Wijermars et al., 2016). Urine metabolomics profiling has proven useful for noninvasively assessing the recovery process in kidney transplant patients (Stenlund et al., 2009), diagnosing and predicting acute cellular rejection in kidney allografts (Suhre et al., 2016), monitoring graft recovery to identify patients whose progress may deviate from expected norms (Calderisi et al., 2013) as well as statistically promising separation of spontaneous tolerant kidney transplanted recipients from healthy subjects (Colas et al., 2022). One of the main challenges in the metabolomics approach to urinary alterations, especially in kidney diseases, remains the proper data handling. There is no unique practice in the normalization of metabolic data from urine to mitigate the effect of possible sample dilution, and widely broad spectra of methods are used: normalization to the sum of spectra or unit area (Calderisi et al., 2013; Stenlund et al., 2009), to osmolality (Suhre et al., 2016), to optical density (Colas et al., 2022) or to the levels of urinary creatinine (Kim et al., 2023).

For this study, to achieve as a homogeneous group as possible, we selectively included patients after primary kidney transplantation, identically treated with immunosuppressants, and on average more than 5 years after transplantation with various severity of kidney dysfunction. We sampled both, morning urine and 24 h collected urine and used the NMR metabolomic approach to determine relative levels of metabolites in urine. In our work, we aimed to reveal the sensitivity of the urinary levels of creatinine to (i) serum creatinine levels and (ii) to allograft function in time of impaired renal functioning in posttransplant patients. We showed the impact of data normalization to creatinine on the metabolic data and carried on the data comparison for morning and 24 h collected urine. In the next part, we discussed the metabolic changes in urine mirroring the worsening of allograft function, which could be considered as indicators of the gradual deterioration of renal function for clinical practice.

## Materials and methods

### Patients

Altogether, 55 adult patients (Caucasians) after the primary transplantation of a kidney from the standard criteria brain-dead donor (SCD), who underwent the transplantation in the Transplant Centre Martin, Slovakia were included in the study. Subjects were characterized by: age at the time of transplantation, sex, presence of biopsy-proven acute rejection (acute cellular rejection (ACR), acute antibody-mediated rejection (AMR)), proteinuria (standard 24 h urine protein test), and finally eGFR-estimated glomerular filtration rate (according to Chronic Kidney Disease Epidemiology Collaboration (CKD-EPI) formula). Acute rejection was confirmed by graft biopsy and classified according to the Banff classification 2019. Donor-specific antibodies were determined by the LUMINEX methodology (positivity was stipulated at ≥ 500 MFI). Standard immunosuppression was identical in the whole group, namely tacrolimus (TAC, Advagraf), mycophenolate sodium (Myfortic), and Prednisone. The group of patients was homogeneous in terms of immunosuppression-average levels of TAC, and daily doses of mycophenolate sodium (Myfortic) and Prednisone were similar (with no significant difference between monitored groups). The average follow-up was 68.4 (SD = 50.7) months after kidney transplantation. Inclusion criteria were as follows: age > 18 years, no active infection, immunosuppression consisting of TAC, MPA, and Prednisone, and primary kidney transplantation from SCD. Exclusion criteria were as follows: age < 18 years, loss to follow-up, active infection, kidney transplantation from extended criteria donor (ECD) or living kidney donor, kidney transplantation in the last 6 months.

A donor with extended criteria means a donor according to the definition of ECD codified in 2002: donors over the age of 60 years without co-morbidities or donors over the age of 50 years with at least two co-morbidities that include blood hypertension, death from cerebrovascular accident, or terminal stage. At the time of urine collection, all patients had clinically determined Cre, eGFR, and proteinuria levels. The stage was assigned by eGFR values according to the generally accepted CKD-EPI formula and KDIGO CKD classification (stage 1 for eGFR 90 and higher, stage 2 for eGFR 60–89, stage 3 for eGFR 30–59, stage 4 for eGFR 15–29, and stage 5 for eGFR less than 15). Patients’ characteristics are summarized in Tables 1, 2. We are not aware of any indication about restricted intake of fluids, ongoing abnormal muscle loss or gain in the selected patients’ group. Donor specific antibodies (DSAs) were detected using the Luminex assay, a multiplex bead-based technology. Serum samples were incubated with a panel of donor antigens. The presence of DSAs was identified through fluorescence, indicating antibodies specific to donor antigens. This method allows for accurate assessment of immunological risk post-transplant. The patients included in our study did not experience treated rejection episodes during the sampling. Any patients with a history of rejection had completed their treatment at least six months prior to the commencement of the study. Patients included in the study were treated in an outpatient clinic, which restricted our ability to directly assess their hydration status. However, we advised patients to consume at least 2000 ml of fluids daily to help maintain consistent hydration levels.

**Table 1:** Characteristics of patients enrolled in the study by stage, including mean values with standard deviation (SD)

Parameter	All	Stage 1	Stage 2	Stage 3	Stage 4	Stage 5
Sample size	55	1	21	21	10	2
Age/years (SD)	52.6 (13.8)	41	48.4 (13.6)	56.8 (13.7)	54.4 (12.7)	42, 48 (2 values)
Sex (F/M)	23/32	0/1	10/11	8/13	5/5	0/2
BMI	28.3 (6.1)	28.1	27.2 (4.7)	27.7 (5.7)	28.9 (5.4)	25.7, 51.2 (2 values)
Serum Cre/µmol/L (SD)	154.6 (79.2)	91	98.3 (14.3)	148.5 (38.2)	237.0 (46.2)	426, 434 (2 values)
eGFR/mL/min/per 1.73 m^2^ (SD)	49.4 (20.8)	91	69.9 (7.1)	43.0 (8.9)	23.7 (2.9)	13, 14 (2 values)
Proteinuria level/g/24 h (SD)	0.847 (1.965)	0.126	0.286 (0.443)	0.985 (2.081)	0.6684 (0.788)	1.17, 11.024 (2 values)

**Table 2:** Characteristics of patients enrolled in the study by history of treated graft rejection, mean values with standard deviation (SD), patients without signs of rejection (CG), with acute cellular rejection (ACR), and with acute antibody-mediated rejection (AMR), donor-specific antibodies (DSA)

Parameter	CG	ACR	AMR
Sample size	35	10	10
Age/years (SD)	56.6 (16.1)	50.0 (12.7)	46.0 (14.2)
Gender (F/M)	14/21	4/6	5/5
BMI	28.6 (5.2)	29.5 (8.3)	26.1 (5.9)
Cre/µmol/L (SD)	123 (43.2)	185.5 (95.9)	234.2 (89.3)
eGFR/mL/min/per 1.73 m^2^ (SD)	58.0 (18.1)	40.6 (18.0)	28.3 (11.2)
Proteinuria level/g/24 h (SD)	0.365 (0.458)	1.222 (2.828)	2.155 (3.076)
DSA presence	0/35	0/10	9/10

### Urine samples

Urine collected over 24 h and morning urine were centrifuged (2000 rpm, 4 °C, 20 min) and aliquots were stored at − 80 °C. After thawing, the urine was centrifuged again at 2000 rpm, at room temperature, for 20 min. For the measurement, 400 µL of centrifuged urine was carefully mixed with 200 µL of a stock solution consisting of (500 mM phosphate buffer, pH-meter reading 7.40, and 0.25 mM TSP-d_4_ (3-(trimethylsilyl)-propionic-2, 2, 3, 3-d_4_ acid sodium salt in deuterated water) as a chemical shift reference in deuterated water). For measurement, 550 μL of the final mixture was transferred into a 5 mm NMR tube.

### Blood plasma samples

Blood samples were collected parallel with the urine samples. The processing to blood plasma, deproteination and and the preparation for the measurement is described in the previous publication (Baranovicova et al., 2022).

### NMR data acquisition

NMR data were acquired on a 600 MHz NMR spectrometer Avance III from Bruker equipped with a TCI (triple resonance) cryoprobe. Initial settings (water suppression frequency, pulse calibration, and shimming) were performed on an independent sample and adopted for measurements. The samples were stored in a Sample Jet at approx. 6 °C before measurement for a maximal time of 3 h. We modified standard Bruker profiling protocols as follows: profiling 1D NOESY with presaturation (noesygppr1d): FID size 64 k, dummy scans 4, number of scans 32, spectral width 20.4750 ppm; COSY with presaturation was acquired for 10 randomly chosen samples (cosygpprqf): FID size 4 k, dummy scans 8, number of scans 1, spectral width 16.0125 ppm; homonuclear*J*-resolved (jresgpprqf): FID size 8 k, dummy scans 16, number of scans 4; profiling CPMG with presaturation (cpmgpr1d, L4 = 126, d20 = 3 ms): FID size 64 k, dummy scans 4, number of scans 512 for urine and 128 for blood plasma samples, spectral width 20.0156 ppm. All experiments were conducted with a relaxation delay of 4 s, and all data were once zero filled. An exponential noise filter was used to introduce 0.3 Hz line broadening before the Fourier transform. Samples were measured at 310 K and randomly ordered for acquisition. The evaluation was performed on cpmg-acquired spectra.

### Data analysis

A chemical shift of 0.000 ppm was assigned to the TSP-d_4_ signal. Spectra were analzyed using an internal metabolite database, an online human metabolome database (www.hmdb.caaccessed in August 2024) (Wishart et al., 2022) and chenomx software (free trial version). For all compounds, the multiplicity of peaks was confirmed in J-resolved spectra, and homonuclear cross peaks were confirmed in COSY spectra. Spectra were binned into bins of 0.001 ppm and integrated according to this resolution. Data were handled as raw data, data normalized to the integral of the creatinine peak at 4.05 ppm, or data normalized to the sum of spectra. Metabolites not having appropriate signals for the evaluations—peak overlap, or with unambiguous peak assignment—were excluded from further evaluation. A table of chemical shifts for metabolites identification is in Supplement.

The null hypothesis of equality of population medians among groups was tested using the nonparametric Kruskal–Wallis test, with Dunn’s post hoc test for pairwise comparison (Matlab, v 2015b). The linear relationships between groups of data were evaluated by Pearsman’s correlation (Origin Pro 2021, academic license).

## Results

In the first step, we compared the levels of urinary creatinine as determined by NMR with the serum creatinine as determined by clinical biochemistry. There was no obvious relation between the data, which was confirmed also by the exact analysis using linear regression. This ended with correlation coefficients of − 0.025 and − 0.034 and with R-square near zero. It is also apparent from data scattering (Figure S1 in Supplement) that the use of any other mathematical model like exponential, logarithmic or polynomic would not lead to the better fitting of the data. No correlations were found also when data were normalized to the sum of spectra, where linear regression finished also with correlation coefficient near zero.

To understand the effect of creatinine normalization on the metabolic data from urine, we analyzed the relations between relative levels of urinary metabolites as raw data and data normalized to the level of urinary creatinine. Strong correlations between raw and normalized levels of metabolites were found for both, morning urine, as well as for 24 h collected urine (Table 3). In the next step, we compared the relative levels of metabolites between morning urine and 24 h pooled urine, and this was also done for raw as well as for creatinine-normalized data (Table 4). Here, slightly better correlations were found for normalized data, although the raw data were also in very good agreement with each other.

**Table 3:** Comparison of relative levels of metabolites between raw data and data normalized to creatinine levels in urine, 24 h pooled urine, and morning urine, (*p* < 0.05 considered significant)

	24 h pooled urine–raw data vs. normalized data		morning urine–raw data vs. normalized data	
	Pearson’s Corr	p-value	Pearson’sCorr	p-value
Alanine	0.74213	4.60E-10	0.7781	4.41E-12
Acetate	0.95245	6.09E-27	0.91433	4.62E-22
Acetone	0.87035	1.09E-16	0.68117	1.44E-08
Pyruvate	0.78776	6.95E-12	0.79241	9.44E-13
Citrate	0.74325	4.20E-10	0.59827	1.77E-06
DMA	0.3944	0.00419	0.34912	0.00967
Carnitine	0.71786	3.05E-09	0.82844	1.07E-14
Hippurate	0.82178	1.47E-13	0.88745	3.97E-19
Formate	0.98071	2.11E-36	0.97397	3.41E-35
1-methylnicotineamid	0.88942	2.78E-18	0.86596	2.84E-17
Phenylalanine	0.69339	1.70E-08	0.69012	7.79E-09
Trigonelline	0.83073	4.63E-14	0.82268	2.34E-14
Fumarate	0.89987	2.76E-19	0.86259	5.19E-17

**Table 4:** Comparison of relative levels of metabolites between 24 h pooled urine and morning urine, done for both, raw data and data normalized to creatinine (*p* < 0.05 considered significant)

	Morning urine vs 24 h collected urine Data normalized to creatinine		Morning urine vs 24 h collected urine Raw data	
	Pearson’s Corr	p-value	Pearson’s Corr	p-value
Creatinine			0.50118	1.22E-04
Alanine	0.77624	1.37E-11	0.79289	1.48E-12
Acetate	0.63566	4.13E-07	0.42257	0.00162
Acetone	0.41583	0.00217	0.33402	0.01451
Pyruvate	0.94397	1.00E-25	0.77511	9.60E-12
Citrate	0.71798	2.08E-09	0.68905	1.17E-08
DMA	0.78674	4.72E-12	0.58333	4.56E-06
Carnitine	0.80935	3.82E-13	0.8421	2.77E-15
Hippurate	0.83472	1.48E-14	0.72197	1.05E-09
Formate	0.9324	9.56E-24	0.82446	3.28E-14
1-methylnicotineamid	0.55736	1.77E-05	0.62378	6.04E-07
Phenylalanine	0.89166	7.73E-19	0.67998	2.15E-08
Trigonelline	0.88271	5.05E-18	0.79916	7.34E-13
Fumarate	0.63779	3.67E-07	0.51233	8.79E-05

As one of the key points of the study, we evaluated the changes in the levels of urinary metabolites regarding the allograft function when expressed by (i) Stage (used data only for stages 2, 3 and 4 which included a sufficient amount of subjects), and (ii) eGFR as a continuous parameter expressing allograft functioning. For both, morning urine and 24 h collected urine, we found no relation of allograft function to urinary creatinine level. In the next, we detected a decrease in urinary level of citrate and acetone with worsening of allograft function for both expression of kidney functioning, by kidney stage (Table 5) and by eGFR (Table 6), The observations for acetone and citrate were comparable for raw as well as for creatinine-normalized data, and for morning urine as well as for 24 h collected urine. We did not detect any other statistically significant correlations or median differences between Stages obtained for any other metabolite, which are all listed in Tables 3, 4. No correlation of urine metabolites levels was found with proteinuria, protein/creatinine ratio when used creatinine values determined by NMR, for both morning urine as well as 24 h pooled urine.

**Table 5:** Statistical evaluation (p-values) of differences in medians in the levels of urinary metabolites for Stages of allograft function in post-transplant patients

	All	Stage 2–3	Stage 2–4	Stage 3–4
	Kruskall Wallis	Post hoc Dun’s test	Post hoc Dun’s test	Post hoc Dun’s test
24 h pooled urine				
Creatinine	0.3798	0.4703	0.3518	0.7016
Acetone	**0.001287**	**0.05996**	**0.0003244**	**0.00311**
Citrate	**0.004139**	**0.01822**	**0.00252**	0.228
Acetone/creatinine	**0.01003**	0.157	**0.002581**	**0.05643**
Citrate/creatinine	**0.006816**	**0.01185**	**0.007138**	0.4463
Morning urine				
Creatinine	0.9719	0.8357	0.9727	0.8486
Acetone	**0.02445**	0.6123	**0.007442**	**0.02399**
Citrate	**0.00334**	**0.06832**	**0.0009324**	**0.05897**
Acetone/creatinine	**0.01665**	0.3804	**0.004287**	**0.03123**
Citrate/creatinine	**0.01305**	0.1063	**0.003926**	0.1047

Bold values indicate significant of *p* value (*p* < 0.05)

**Table 6:** Pearson’s correlations of raw levels of urinary metabolites and the allograft functioning expressed by eGFR for both, morning urine and 24 h collected urine, data shown for creatinine, and acetone and citrate

	Morning urine		24 h collected urine	
	Pearson Corr	p-value	Pearson Corr	p-value
Creatinine	0.12631	0.34914	0.15733	0.25132
Acetone	0.26453	**0.04676**	0.47581	**4.257e-04**
Citrate	0.41374	**0.00138**	0.4528	**8.49e-04**
Acetone/creatinine	0.26188	**0.04909**	0.4655	**5.77e-4**
Citrate/creatinine	0.41035	**0.00152**	0.4353	**0.00141**

Bold values indicate significant of *p* value (*p* < 0.05)

Additionally, we checked the correlations of urinary metabolite levels with eGFR for data normalized to the sum of spectra. Here, we found two correlations with eGFR: citrate level p-value = 0.0024 and r = 0.41, acetone level p-value 0.0108 and r = 0.35 for morning urine and citrate level p-value = 0.00033and r = 0.47, acetone level p-value 0.000023 and r = 0.54 for 24 hours pooled urine (data not included in the Tables). No further correlations were found.

**Fig. 1:**
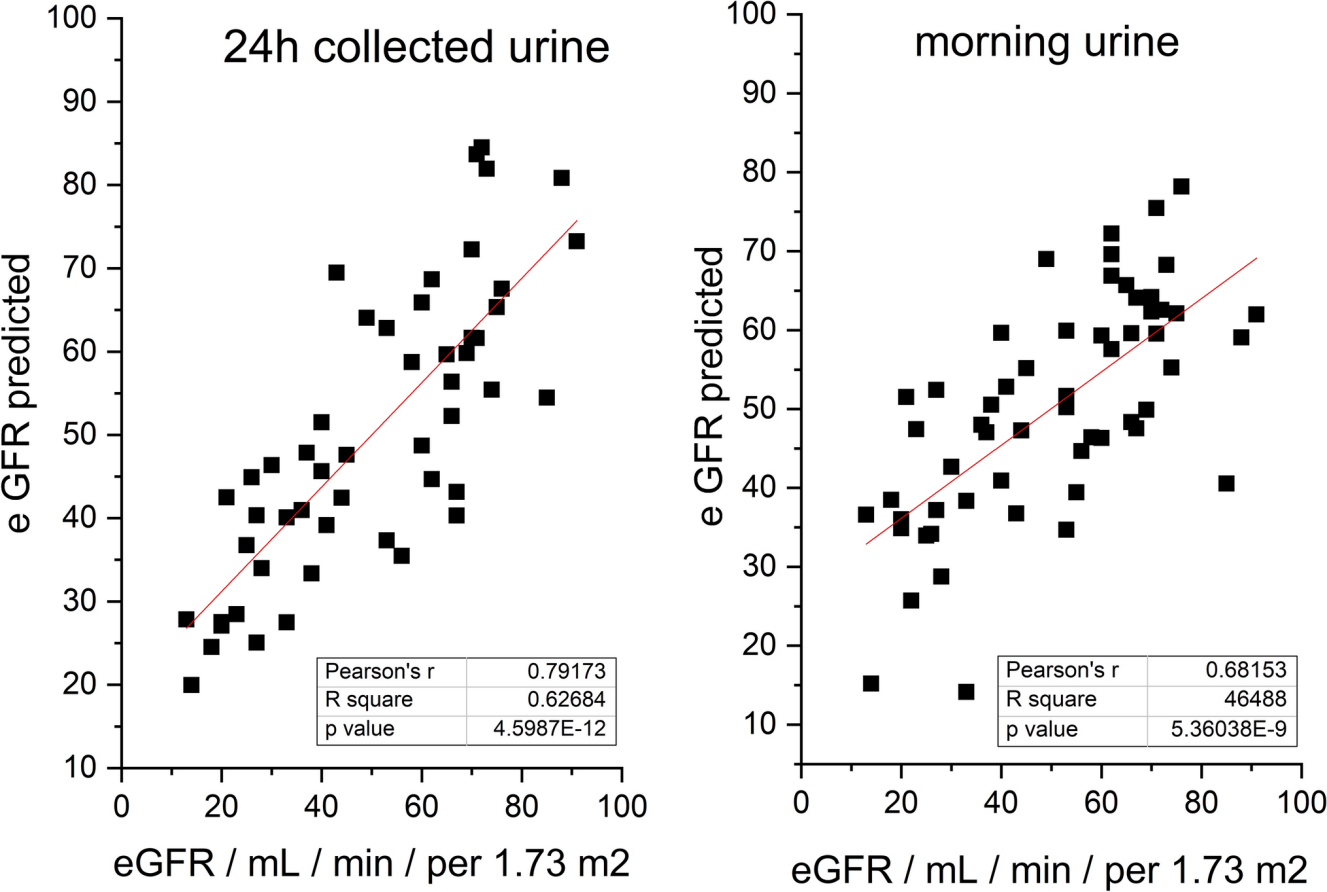
Results from Pearson’s correlation following multilinear regression analysis, where urinary concentrations were used as independent variables; predicted vs. measured eGFR: 24 h pooled urine: p-value = 4.60 × 10^−12^ and r = 0.79, morning urine: p-value = 5.36 × 10^−9^ and r = 0.68)

We performed a multiple linearregression analysis (as a simple and well understable regression tool) where it was of interest if a linear combination of urinary levels of selected metabolites could predict the allograft function expressed by the clinically determined eGFR. For the regression model, we used all determined urinary metabolites as independent variables. The predicted vs. measured eGFR values correlated as follows: for 24 hours pooled urine: p-value = 4.6 × 10^−12^ and r = 0.79 and morning urine p-value = 5.36 × 10^−9^ and r = 0.68 (Fig. 1).

### Blood plasma data

To enhance the understanding of the patient’s metabolic profile, we used metabolic data from blood plasma (Table 7), data collected on the same cohort (Baranovicova et al., 2022). No significant correlations were found for the metabolites lactate, BCAAs (leucine, isoleucine, valine), glucose, pyruvate, 3-hydroxybutyrate, and creatine.

**Table 7:** Pearson’s correlations of serum creatinine (Cre), eGFR and proteinuria with relative metabolites level in blood plasma, determined by NMR spectroscopy

	Cre (µmol/L)		eGFR (mL/min/per 1.73m^2^)		proteinuria (g/24 h)	
	r	p-value	r	p-value	r	p-value
Alanine	0.28	**0.044**	− 0.11	0.40	0.02	0.86
Citrate	0.10	0.43	− 0.29	**0.031**	− 0.04	0.76
Phenylalanine	0.45	**0.00044**	− 0.46	**0.00030**	− 0.06	0.62
Tyrosine	− 0.26	0.05	0.18	0.16	− 0.20	0.12
Glutamine	0.48	**0.00016**	− 0.39	**0.0026**	0	0.98
Ketoleucine	− 0.14	0.27	0.30	**0.02**	− 0.36	**0.0055**
Ketoisoleucine	− 0.15	0.26	0.23	0.08	− 0.28	**0.033**
Ketovaline	− 0.20	0.12	0.26	0.05	− 0.43	**0.0010**
Creatinine	0.48	**0.00016**	− 0.83	**3.4E-15**	0.42	**0.0013**
Proline	0.45	**0.00049**	− 0.33	**0.011**	0.02	0.84
Histidine	0.66	**3.6E-08**	− 0.39	**0.0031**	0.13	0.32

Bold values indicate significant of *p* value (*p* < 0.05)

## Discussion

### Urinary level of creatinine level in patients with changed allograft function

**Fig. 2:**
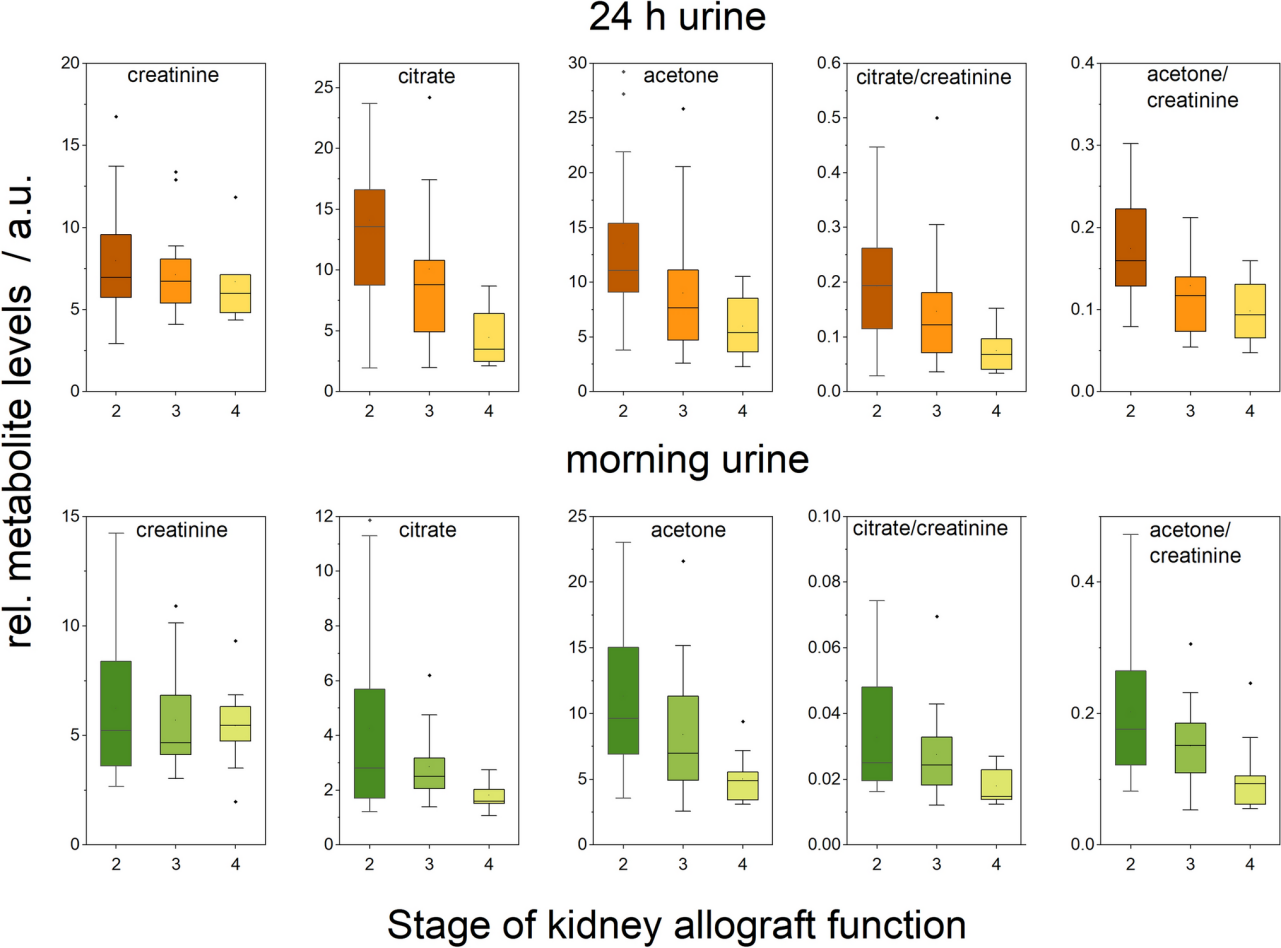
Metabolites levels in urine in relation to the stage of allograft function

Creatinine is the final product of muscle energy metabolism and it is believed to be excreted from the body by the renal way without further metabolic transformation in that amount in which produced. As the kidneys are responsible for creatinine clearance, serum creatinine levels are widely used as a parameter for estimation of renal function and serves as an input variable for the approximation of GFR, the index of kidney function. The implicit assumption is that urinary creatinine excretion is constant across and within healthy individuals; however, creatinine values in urine depend on diet and muscle mass, activity, and, of course, hydration status. Urinary albumin or other markers of kidney injury are frequently reported as a normalized ratio to urinary creatinine concentration, however, it makes the urinary quantity of any parameter dependent not only on its excretion rate but also on the urinary flow rate. If we follow the assumption of decreased creatinine urine excretion in conditions of poor renal allograft function (Waikar et al., 2010), all parameters normalized to urine creatinine would be amplified with decreasing glomerular filtration rate, which coulddistort the observation, especially when the parameter itself is not subject to large changes. In subjects with nonsteady-state conditions such as chronic kidney disease (CKD), the results by (Di Micco et al., 2013) showed decreasing creatinine levels in 24 h collected urine with worsening of CKD stage, more pronounced for male subjects. This result would be in agreement with the theoretical simulations done by (Waikar et al., 2010), which proposed an inverse relation between serum creatinine and urinary creatinine in the conditions of impaired kidney function. However, contrary to the observations presented by (Di Micco et al., 2013) and theoretical assumptions by (Waikar et al., 2010), the data summarized by Jain from almost 40,000 participants with CKD did not show any convincing relation between urinary and serum creatinine levels (Jain, 2016). In summary of the mentioned study, the circulating level of creatinine increased with the CKD stage (naturally, as they are not independent parameters), however, there were minimal differences in urinary creatinine levels for CKD stages 1, 2, 3a, 4 and 5 with one decline for stage 3b, which was the only significantly different observation. In our study on a set of patients after kidney transplantation with various severity of allograft dysfunction, likewise, we did not observe any relation between urinary and serum creatinine levels, as well as no statistical differences between urinary creatinine levels in the stages 2, 3 and 4 of allograft function were detected (Fig. 2, Table 6). This applies for both, morning urine as well as for 24-h collected urine.

The observed insensitivity of urinary creatinine levels to allograft efficiency makes urine creatinine level act as an algebraic variable independent of renal function. This naturally contributes to the good agreement of urinary levels of metabolites derived from raw and creatinine normalized data (Table 3). This finding, of course, does not imply the use of normalization to creatinine as the only proper practice in conditions of altered renal function, especially when not having a simple and clear explanation which would clarify why the experimental data show the trend different from expectations.

In this study, we used data from 55 patients with various severity of allograft dysfunction after transplantation, which could be considered a limitation. However, a very similar result was obtained in a very comprehensive study (Jain, 2016) which may be believed to present unquestionable results. The analytical method used in this study is known for its robustness (Moco, 2022), patients were chosen with a high effort to achieve high homogeneity regarding their metabolic conditions including treatment. Maybe the direction for understanding may be in one other study by (Tynkevich et al., 2015), where, again, no relation of urinary creatinine to eGFR was observed in CKD subjects, however, there was detected a certain relation of urinary creatinine to mGFR, which is calculated on iodthalamate clearance and provides a more precise measurement of kidney function.

From the other side, there is evidence that urinary creatinine is formed not only by glomerular filtration from blood but is also secreted by the proximal tubule. This is the reason why the calculated creatinine clearance (which uses urinary creatinine in the numerator and serum creatinine in the denominator) may overestimate true GFR by 10–20% (Garimella et al., 2021).The difference between measured creatinine clearance and measured GFR was suggested to represent the clearance of creatinine due to tubular secretion (Garimella et al., 2021). The interesting behavior of the indexes describing renal function was observed, when with diminishing the renal function the GFR and creatinine clearance decreased, however, the longstanding observation is that the creatinine clearance/GFR ratio increases as GFR decreases in CKD patients. It was believed that it is the effect of (i) measurement error (Zhang et al., 2016), which was opposed by data collected by (Zhang et al., 2020), or (ii) the fact that in renal diseases accompanied by diminished GFR, the contribution of tubular creatinine secretion to creatinine clearance may be dramatically increased (Ciarimboli et al., 2012; Shemesh et al., 1985). The data by Garimella et, al., however, showed a contrasting trend, namely, the slight decrease of tubular creatinine secretion with decreasing mGFR (Garimella et al., 2021), although the data points were rather scattered with Pearson’s r = 0.3. Returning to the data obtained in our study, these are consistent with multiple observations of an increasing creatinine clearance/GFR ratio in times of renal malfunction. It is questionable if the clarification can be simply based on the alteration of tubular secretion, which (whether accelerated or suppressed) would affect also the serum creatinine values, as an input variable for creatinine clearance or eGFR. At the level of further hypotheses, which could contribute to the result, although rather to a lesser extent, remain the possible factors such as urine dilution, creatinine stability over the sampling time, subject properties: catabolic and anabolic processes in muscles and tissues, muscle mass, then the in the case of posttransplant patients also the duration of cold ischemia, donor’s characteristics and many others, what could be subjects for further clarifications.

### Metabolic changes in urine depending on allograft function

In our work, we observed a decrease in urinary levels of citrate and acetone with worsened allograft function for both urine types, morning urine as well as 24 h collected urine (Tables 5, 6, Fig. 2). Citrate is a crucial metabolite in the energy-producing Krebs cycle. In intact conditions, it is freely filtered by the kidney glomerulus, with 65–90% reabsorbed in the proximal tubule, leaving about 10–35% to be excreted in the urine (Hamm, 1990; Hering-Smith & Hamm, 2018). Citrate also serves as a metabolic fuel for the kidney and as an excellent chelating agent acts as a natural inhibitor of calcium kidney stones (Hering-Smith & Hamm, 2018). Notably, citrate can be metabolized by the kidneys into HCO_3_^−^, making it a potential base and suggesting a possible, though not well-studied, role in acid–base balance (Hering-Smith & Hamm, 2018). The study by (Gianella et al., 2021) proposed possible reasons for the fall in urinary citrate (citrate/creatinine ratio) in subjects with lowered kidney function to be (i) the fall of plasma citrate (theoretically, without the data), (ii) increased tubular reabsorption of citrate, or (iii) filtered load from decreased GFR. In our study, we observed increased levels of circulating citrate with the loss of allograft function (Table 7), what would contradict the first proposed mechanism by (Gianella et al., 2021). It seems that with increasing renal malfunction (as observed in patients after kidney transplantation), the levels of blood citrate and urinary citrate are going in opposite directions. One of the explanations for this behavior could be the accelerated citrate reabsorption in the proximal tubules of the kidney in times of altered kidney function as a response to the changes in acid–base balance. However, having the metabolic data from the same subjects, the monitored group of patients did not show any changes (or correlations with eGFR) neither in ‘metabolic acids’ such as lactic acid or pyruvic acid nor glucose or 3-hydroxybutyratea ketone body representative (Table 7). Monitored patients showed the ASTRUP (a diagnostic test used to assess acid–base status) within the normal range, or at most their condition was compensated by bicarbonate. Therefore, the increased reabsorption of citrate as an adjustment to the shifted pH balance is rather unlikely.

Alterations in citrate levels can both cause as well as result from mitochondrial dysfunction. This typically precedes but can also be in a bidirectional relationship with events such as ischemia–reperfusion injury, oxidative stress, inflammation, and allograft rejection. Although mitochondrial dysfunction is not the primary cause of allograft dysfunction, it plays a significant contributory role in the development and progression of kidney dysfunction following transplantation. The discussion related to energy production within the mitochondria could be directly supported by known levels of other metabolites than citrate. Due to significant peak overlap in NMR spectra, we did not evaluate the succinate signal, although it was clearly visible. With decline of mitochondrial function, cells increase the rate of glycolysis as a compensatory mechanism to meet cellular energy demand. This may lead to enhanced lactate production, which, as already mentioned above, was not observed in the selected patient cohorts in circulation as related to allograft function. We also did not observe any relation of urinary or circulating pyruvate to allograft function expressed by eGFR, which would indicate the increasing incapacity/inability of damaged mitochondria to efficiently process pyruvate. However, mitochondrial dysfunction can exist even if pyruvate, lactate, and glucose levels remain unaltered, especially in cases where compensatory metabolic pathways are sufficient to maintain cellular energy homeostasis. Based on our limited data, a gradual/progressive deterioration of mitochondrial functions with decreasing allograft function in our patients cannot be verified nor excluded, as a systematic change. Taking together all the mentioned facts, we consider the interpretation derived from decreased citrate filtration rate in the time of affected function of the glomerulus, which is rational for the increased levels of citrate in circulation (Table 7) as decreased levels of citrate in urine (Table 6) for acceptable.

The second metabolite whose urinary levels were found to be sensitive to allograft function was acetone. It is naturally produced in the body in small amounts, however, under certain conditions such as ketosis or ketoacidosis its level may increase. As already mentioned, the group of patients included in this study did not show any signs of turnover or any shift in energy metabolism. Therefore, we can assume relatively constant acetone production in these patients, although the exact data are not available. A significant portion of acetone is expelled through the lungs, however, acetone is also filtered out by the kidneys and excreted in the urine with minimal reabsorption. Taken together, lowered levels of acetone, similar to citrate in urine in time of decreased allograft function are likely to be related to ineffective filtration.

In this work, we did not find any relation between the levels of urinary metabolites and proteinuria, or the protein/creatinine ratio (creatinine values were used as determined by NMR). This suggests that urinary metabolite levels may not directly reflect kidney damage aspect. Proteinuria as a non-specific indicator is associated with structural damage to the kidneys and does not necessarily correlate strongly with the actual functional impairment.

It remains the question, why just the levels of these 2 metabolites from the group of 14 evaluated metabolites are selectively sensitive to estimated glomerular filtration and not the others, which is not safe to answer without further data. It is obvious that having the data about metabolic changes in circulation was very advantageous and offered a more comprehensive view into the metabolic state of an organism. Equally, altered kidney functioni itself may contribute to further metabolic modifications. This fact emphasizes the need for more comprehensive metabolic studies since every alteration is the result of the complex mutual biochemical pathways in the comprehensive inter-organ metabolic exchange and communication.

Lastly, the levels of urinary metabolites, as determined by NMR spectroscopy, in combination showed a particular prediction ability to eGFR, slightly better for the 24 h pooled urine (Fig. 1). Although this is not enough in itself for use in clinical practice, urinary metabolomics can serve as an important supplement to blood plasma metabolomics, as we showed previously (Baranovicova et al., 2022) and together they may present a useful tool forthe prediction of metabolic allograft function in patients after kidney transplantation.

Notes on the study and its limitations.

(i) We acknowledge the limited number of metabolites evaluated. In NMR, the relative concentration of a metabolite is linearly related to the integral of its signal, which must be integrated from the baseline for accurate data. Metabolic changes detectable by NMR are typically small (20–30%) compared to protein/enzymes/miRNA expression. Therefore, signals affected by overlap may lead to inaccurate evaluations. Some urine samples also contained unknown signals, complicating spectrum deconvolution. In this work, we used an untargeted approach focusing on metabolites with well-defined peaks, minimally affected by other signals. While this approach may sacrifice some information, it ensures the accuracy and reliability of the data evaluated (for details, please see Supplement File).
(ii) It should also be mentioned that around 40% of the patients included experienced rejection episodes in the history (Table 2). Rejection-related tubular dysfunction in kidney transplant recipients can often be reversed with early treatment. However, if permanent damage occurs, the tubules’ ability to filter, reabsorb, or secrete metabolites is impaired, leading to elevated concentrations of substances in the urine. Tubular dysfunction can also disrupt the regulation of electrolytes such as sodium, potassium, and calcium, which can, in turn, affect the concentration and reabsorption of metabolites that rely on electrolyte gradients for proper renal function. Tubular dysfunction can indirectly affect eGFR by altering how creatinine is processed, which may distort the true glomerular filtration rate.
(iii) In this work, we used metabolomic data on blood plasma to support the discussion. These data are comprehensively discussed in our previous work (Baranovicova et al., 2022).

## Conclusion

In our study on patients after kidney transplantation with varying degrees of allograft dysfunction, we did not detect any relation between urinary and serum creatinine levels. Accordingly, no differences were observed in urinary creatinine levels across stages 2, 3, and 4 of renal function, nor was any relation found between urinary creatinine levels and eGFR. This observation, which is not in line with primary expectations was discussed and led to the support the opinion that creatinine clearance, especially urinary creatinine values, should be critically reconsidered when using as a parameter assessing the glomerular filtration in casef ofof restricted renal function.The observed insensitivity of urinary creatinine levels to allograft efficiency suggests that urine creatinine levels actsas variable independent of renal function, what explains the uniformity of the results seen in both from raw and creatinine-normalized data. We also found very good overlap for results obtained for morning urine samples and 24-h urine collections. The urinary levels of citrate and acetone were found to be sensitive indicators of allograft function, and the combined urinary metabolite levels demonstrated a strong predictive value for kidney function, with highly significant p-values of 4.6 × 10^−12^ for 24-h pooled urine and 5.36 × 10^−9^ for morning urine samples. Although the raw data are mostly discussed, the data normalization to the sum of spectra led to comparable results and conclusion. As our study follows the study on blood plasma, having information on metabolic changes in circulation provided a more complete understanding of the organism’s metabolic state, with broader options for results discussion. This highlights the importance of conducting more comprehensive metabolic studies, as each alteration results from complex, interconnected biochemical pathways that facilitate metabolic inter-organ exchange and communication.

## Supplementary Information

Below is the link to the electronic supplementary material.

Supplementary file1 (DOCX 382 KB)

## Data Availability

All data concerning the study, including raw NMR spectra and data evaluated, are available on a reasonable request: eva.baranovicova@uniba.sk.
