# Supplementary material for: Assessment of metabolites in urine in post-kidney transplant patients: insights into allograft function and creatinine clearance
Source: Metabolomics. 2025 Mar 27;21(2):44. doi: 10.1007/s11306-025-02246-y (PMC11950123; doi:10.1007/s11306-025-02246-y)
Supplement: Supplementary file 1 — Supplementary file1 (DOCX 382 KB) [file 11306_2025_2246_MOESM1_ESM.docx]

Supplement

Table S1. Chemical shifts (in ppm) and multiplicities (s—singlet, m—multiplet) for the pool of metabolites identified in urine.

| **metabolite** | **NMR peak assignment** |
| --- | --- |
| **1-methylnicotineamid** | 8.18 (m), 8.90(m), 8.96 (m), 9.22(s) |
| **acetate** | 1.92 (s) |
| **acetone** | 2.23 (s) |
| **alanine** | 1.48 (d; J=7.30), 3.78 (q, J = 7.23) |
| **carnitine** | 2.39 (m), 2.43 (m), 3.19 (s), 3.41 (m), 3.45 (m), 4.56 (m) |
| **citrate** | 2.54 (d; J = 15.1), 2.67 (d; J = 15.1) |
| **creatinine** | 3.05 (s), 4.07 (s) |
| **DMA** | 2.695 (s) |
| **formate** | 8.46 (s) |
| **fumarate** | 6.52 (s) |
| **hippurate** | 3.95 (m), 7.55 (m), 7.64 (m), 7.84 (m) |
| **phenylalanine** | 7.33(m), 7.69 (m), 7.43 (m) |
| **pyruvate** | 2.38 (s) |
| **trigonelline** | 8.07 (m), 8.83 (m), 8.84 (m), 9.12 (s) |


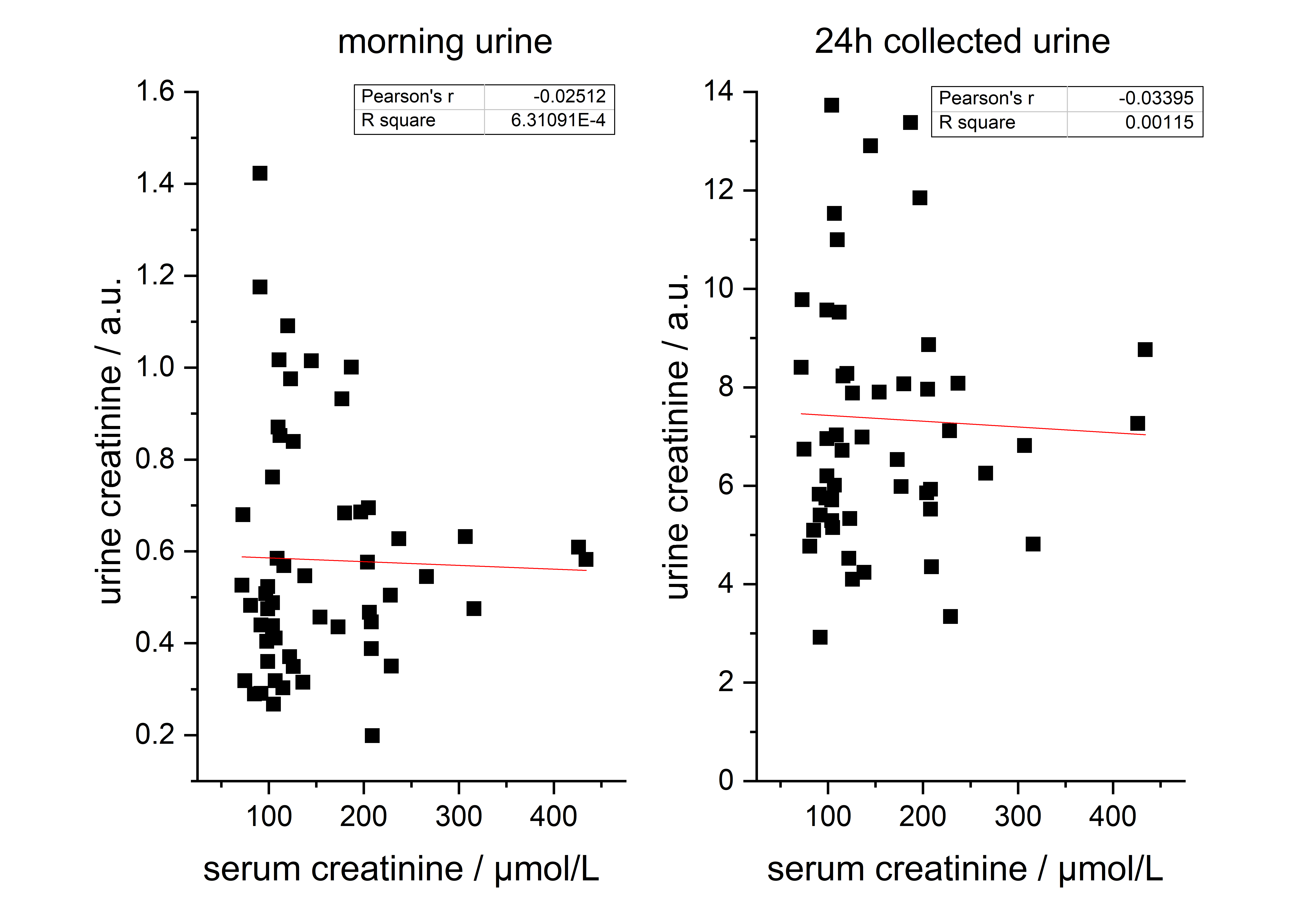


Figure S1. Relation between serum creatinine levels and urinary creatinine levels in patients after kidney transplantation with various allograft functions.

**Note to the reduced number of metabolites determined:**

We are aware of the reduced number of metabolites evaluated. In NMR, the relative concentration of a metabolite is linearly related to the integral of the assigned signal. To obtain appropriate data, a signal must be integrated from the baseline. In the figure below, we show the signal of creatinine as an example of a signal that is minimally affected by signals from other co-metabolites, and the integration of this signal offers reliable data. Below, we also show the signal of a lactate doublet, which is well-recognized and confirmed in JRES as well as COSY spectra. However, the integral value of this signal is affected by overlapping signals, and therefore, it does not represent a reliable value. Finally, we show the signal of TSP-d_4_, which is used as a chemical shift reference as well as a ‘control signal’ for assessing the quality of NMR shimming. Throughout the entire acquisition process, we ensured that the half-width of this signal remained below 1.0 Hz to maintain high-quality spectra.

It is well known that metabolic changes detectable by NMR are seldom greater than approximately 20-30%, compared to protein/enzymes/miRNA expression, which can show altered values by several multiples. Therefore, integrating signals that are affected or overlapped may not accurately represent the true values and can distort the evaluation, results, and conclusions of the study. Additionally, some urine samples from patients contained, alongside the well-known signals, unknown signals that were not unambiguously assigned, making the spectra deconvolution process more difficult.

In summary, we employed an untargeted approach with a limited set of metabolites, focusing on metabolites with well-recognizable peaks that were not, or minimally, affected by other signals. We consider this procedure to be correct, even at the cost of losing some information, which could be affected by error and therefore may not reflect the true reality.


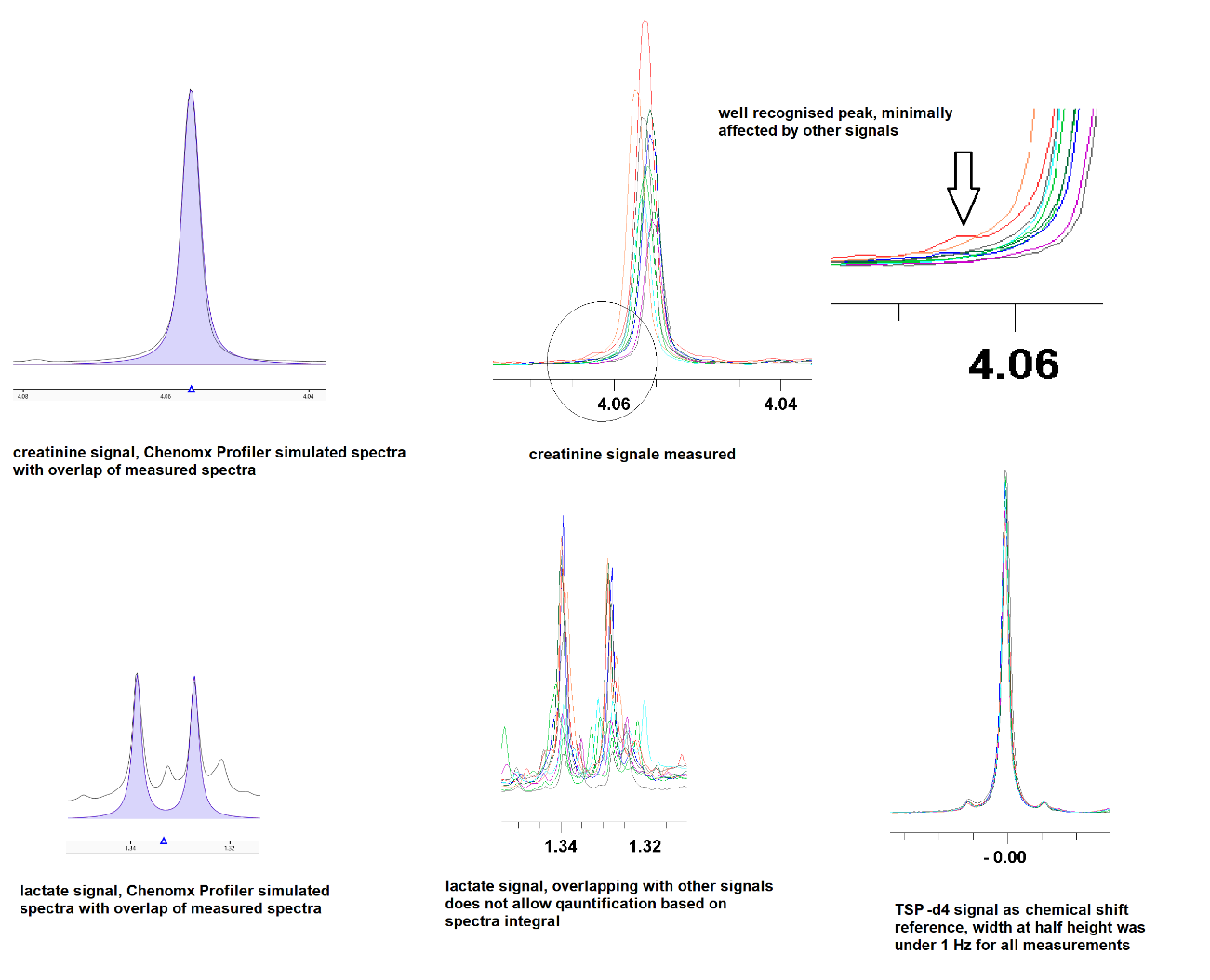


Figure S2 NMR signals for selected metabolites.
